# Online Coregularization for Multiview Semisupervised Learning

**DOI:** 10.1155/2013/398146

**Published:** 2013-09-08

**Authors:** Boliang Sun, Guohui Li, Li Jia, Kuihua Huang

**Affiliations:** College of Information System and Management, National University of Defense Technology, Changsha, Hunan 410073, China

## Abstract

We propose a novel online coregularization framework for multiview semisupervised learning based on the notion of duality in constrained optimization. Using the weak duality theorem, we reduce the online coregularization to the task of increasing the dual function. We demonstrate that the existing online coregularization algorithms in previous work can be viewed as an approximation of our dual ascending process using gradient ascent. New algorithms are derived based on the idea of ascending the dual function more aggressively. For practical purpose, we also propose two sparse approximation approaches for kernel representation to reduce the computational complexity. Experiments show that our derived online coregularization algorithms achieve risk and accuracy comparable to offline algorithms while consuming less time and memory. Specially, our online coregularization algorithms are able to deal with concept drift and maintain a much smaller error rate. This paper paves a way to the design and analysis of online coregularization algorithms.

## 1. Introduction

Semi-supervised learning (S^2^L) is a relatively new subfield of machine learning which has become a popular research topic throughout the last two decades [1–6]. Different from standard supervised learning (SL), the S^2^L paradigm learns from both labeled and unlabeled examples. In this paper, we investigate the online semi-supervised learning (OS^2^L) problems with multiple views which have four features: (1) data is abundant, but the resources to label them are limited; (2) data arrives in a stream and cannot store them all; (3) the target functions in each view agree on labels of most examples (compatibility assumption); (4) the views are independent given the labels (independence assumption). 

OS^2^L algorithms take place in a sequence of consecutive rounds. On each round, the learner is given a training example and is required to predict the label if the example is unlabeled. To label the examples, the learner uses a prediction mechanism which builds a mapping from the set of examples to the set of labels. The quality of an OS^2^L algorithm is measured by the cumulative loss it makes along its run. The challenge of OS^2^L is that we do not observe the true label for unlabeled examples to evaluate the performance of prediction mechanism. Thus, if we want to update the prediction mechanism, we have to rely on indirect forms of feedback. 

Lots of OS^2^L algorithms have been proposed in recent years (see a survey in [7, 8]). A popular idea is defining an instantaneous risk function and decreasing its value in an online manner to avoid optimizing the primal semi-supervised problem directly [9–11]. References [12–14] also treat the OS^2^L problem as online semi-supervised clustering in that there are some *must-links *pairs (in the same cluster) and *cannot-links *pairs (cannot in the same cluster), but the effects of these methods are often influenced by “bridge points” (see a survey in [15]).

Coregularization [2, 16] is a method of improving the generalization accuracy of SVMs [17] by using unlabeled data in different views. Multiple hypotheses are trained in coregularization framework and are required to make similar predictions on any given unlabeled example. Moreover, theoretical investigations demonstrate that the coregularization approach reduces the Rademacher complexity by an amount that depends on the “distance” between the views [18, 19]. Unfortunately, basic offline coregularization algorithms are still unable to deal with long-playing large-scale OS^2^L problems directly because of the constraint of time and memory.

In this paper, we introduce a novel online coregularization framework for the design and analysis of new OS^2^L algorithms. Since decreasing the primal coregularization objective function is impossible before obtaining all the training examples, we propose a Fenchel conjugate transform to increase the dual problem incrementally. The existing online coregularization algorithms in previous work can be viewed as an approximation of the dual ascending process based on gradient ascent. New online coregularization algorithms are derived based on the idea of ascending the dual function more aggressively. We also discuss the applicability of our framework to the settings where the target hypothesis is not fixed but drifts with the sequence of examples.

To the best of our knowledge, the closest prior work is proposed by de Ruijter and Tsivtsivadze [10]. Their method defines an instantaneous regularized risk function using part of examples to avoid optimizing the primal coregularization problem directly. The learning process is based on convex programming with stochastic gradient descent in kernel space. The update scheme of this work can also be derived from our online coregularization framework. 

The rest of the paper will be organized as follows. In Section 2 we begin with a primal view of multiview semi-supervised learning problem based on coregularization. In Section 3 our new framework for designing and analyzing online coregularization algorithms is introduced. Next, in Section 4, we demonstrate that the existing online coregularization algorithms can be derived from our framework using gradient ascent. New online coregularization algorithms are derived based on aggressive dual ascending procedures in Section 5. Experiments and analyses are in Section 6. In Section 7, conclusions and possible extensions of our work are given.

## 2. Basic Problem Setting

Our notation and problem setting are formally introduced in this section. The italic lower case letters refer to scalars (e.g., *α* and *w*), and the bold letters refer to vectors (e.g., **ω** and ***λ***). (**x**_*t*_, *y*_*t*_, *σ*_*t*_) denotes the *t*th training example, where **x**_*t*_ = (**x**_*t*_^(1)^, **x**_*t*_^(2)^,…, **x**_*t*_^(*s*)^) is seen in *s* views with **x**_*t*_^(*i*)^ ∈ *X* ^(*i*)^ (*i* ∈ {1,2,…, *s*}), *y*_*t*_ is its label, and *σ*_*t*_ is a flag to determine whether the label can be seen. If *σ* = 1, the example is labeled; and if *σ* = 0, the example is unlabeled. The hinge function is denoted by [*a*]_+_ = max⁡{*a*, 0}. 〈**ω**, **x**〉 denotes the inner product between vectors **ω** and **x**.

In most cases, the hypotheses used for prediction come from a parameterized set of hypotheses, *ℋ* = {*h*_**ω**_ : **ω** ∈ *V*}, where *V* is a subset of a vector space. For simplicity and concreteness, we focus on semi-supervised binary classification problems in two views in this paper. Consider an input sequence (**x**_1_^(1,2)^, *y*_1_, *σ*_1_), (**x**_2_^(1,2)^, *y*_2_, *σ*_2_),…, (**x**_*T*_^(1,2)^, *y*_*T*_, *σ*_*T*_), where **x**_*t*_^(1,2)^ = (**x**_*t*_^(1)^, **x**_*t*_^(2)^), *y*_*t*_ ∈ {−1, +1}, and *σ*_*t*_ ∈ {0,1} (*t* ∈ {1,2,…, *T*}). The goal is to learn the function pair *f* = (*f* ^(1)^, *f* ^(2)^), where *f* ^(1)^ = 〈**ω** ^(1)^, **x** ^(1)^〉 and *f* ^(2)^ = 〈**ω** ^(2)^, **x** ^(2)^〉 are linear classifiers in two views, respectively. To learn max-margin decision boundaries, the multiview S^2^L problem based on coregularization [16] can be written as minimizing:

(1) J(ω(1),ω(2)) =12γ1||ω(1)||2+12γ2||ω(2)||2  +∑t=1Tσt([1−yt〈ω(1),xt(1)〉]++μ[1−yt〈ω(2),xt(2)〉]+)  +∑t=1T(1−σt)γcd(〈ω(1),xt(1)〉,〈ω(2),xt(2)〉),

where *γ*_1_ and *γ*_2_ are trade-off parameters for complexity functions in the two views, *μ* is a real-valued parameter to balance data fitting, *γ*_*c*_ is the coupling parameter that regularizes the pair towards compatibility using unlabeled data, and *d*(·, ·) is the distance function which measures the difference between the predictions for the same example and composes the multiview coregularizer. 

In previous approaches based on coregularization [16, 19], the distance function *d*(·, ·) is often defined as a square function:

(2) d(〈ω(1),xt(1)〉,〈ω(2),xt(2)〉)=(〈ω(1),xt(1)〉−〈ω(2),xt(2)〉)2.

The distance function is defined as an absolute function (using *l*_1_ norm) in this paper (this idea is also adopted by Szedmak and Shawe-Taylor [18] and Sun et al. [20]):

(3) d(〈ω(1),xt(1)〉,〈ω(2),xt(2)〉)=|〈ω(1),xt(1)〉−〈ω(2),xt(2)〉|.

**Figure 1:**
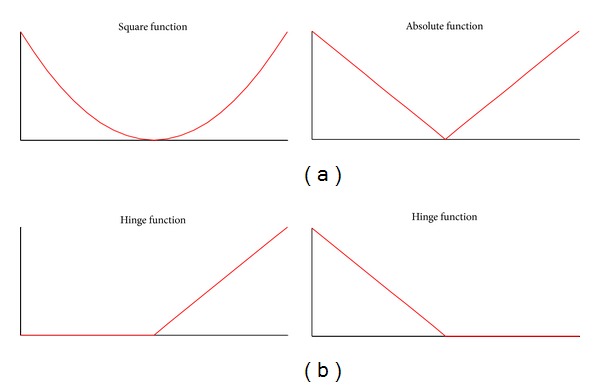
Two different distance functions and hinge functions. The top plane shows the two different distance function. The bottom plane shows that the absolute function |**x**| can be decomposed into two hinge functions [**x**]_+_ and [−**x**]_+_.

Furthermore, (3) is composed of two hinge functions (see Figure 1 for an illustration)

(4) d=[〈ω(1),xt(1)〉−〈ω(2),xt(2)〉]++[〈ω(2),xt(2)〉−〈ω(1),xt(1)〉]+.

In the next section, we will show that the online coregularization problem can be discussed in the dual form of (1) more easily and directly while using the absolute distance function.

Denote the *instantaneous loss* on round *t* as

(5) gt(ω(1),ω(2))=σt([1−yt〈ω(1),xt(1)〉]+   +μ[1−yt〈ω(2),xt(2)〉]+) +(1−σt)γc|〈ω(1),xt(1)〉−〈ω(2),xt(2)〉|,

where *t* ∈ {1,2,…, *T*}. We thus get a simple version of (1) using (5):

(6) J(ω(1),ω(2))=12γ1||ω(1)||2+12γ2||ω(2)||2+∑t=1Tgt(ω(1),ω(2)).

The minimization problem of (6) in an online manner is what we consider in the rest of this paper. 

## 3. Online Coregularization by Ascending the Dual Function

In this section, we propose a unified online coregularization framework for multi-view semi-supervised binary classification problems. Our presentation reveals how the multiview S^2^L problem based on coregularization in Section 2 can be optimized in an online manner.

Before describing our framework, let us recall the definition of Fenchel conjugate that we use as a main analysis tool in this paper (see the appendix for more details). The Fenchel conjugate of a function *f* : dom⁡*f* → ℝ is defined as

(7) f∗(λ)=sup⁡{〈λ,ω〉−f(ω):ω∈dom⁡f}.

As shown in (7), the Fenchel conjugate is defined only for single variable function in former convex analysis. We extend the definition of Fenchel conjugate to multivariables functions for solving online multiview S^2^L problem in this paper. 

The Fenchel conjugate of a multivariables function *f*(**ω** ^(1)^, **ω** ^(2)^,…, **ω** ^(*s*)^) (**ω** ^(1)^ ∈ *X* ^(1)^, **ω** ^(2)^ ∈ *X* ^(2)^,…, **ω** ^(*s*)^ ∈ *X* ^(*s*)^) is defined as

(8) f∗(λ(1),λ(2),…,λ(s))  =sup⁡{∑i=1s〈λ(i),ω(i)〉−f(ω(1),ω(2),…,ω(s))  :     ω(1)∈X(1),ω(2)∈X(2),…,ω(s)∈X(s)}.

Specially, the Fenchel conjugate of hinge functions is a key to transfer coregularization from offline to online in this paper.

**Proposition 1**

Let *f*(**ω** ^(1)^, **ω** ^(2)^) = [*b*−(〈**ω** ^(1)^,**x** ^(1)^〉−〈**ω** ^(2)^,**x** ^(2)^〉)]_+_, where **ω** ^(1)^ ∈ *X* ^(1)^, **ω** ^(2)^ ∈ *X* ^(2)^, and *b* ∈ ℝ. The Fenchel conjugate of *f*(**ω** ^(1)^, **ω** ^(2)^) is

(9) f∗(λ(1),λ(2))={−αbif  λ(1)=−αx(1),  λ(2)=αx(2),where  α∈[0,1]∞otherwise.

**Proof**

We first rewrite the *f*(**ω** ^(1)^, **ω** ^(2)^) as follows:

(10) f(ω(1),ω(2))=[b−(〈ω(1),x(1)〉−〈ω(2),x(2)〉)]+=max⁡α∈[0,1]⁡α(b−(〈ω(1),x(1)〉−〈ω(2),x(2)〉)).

Based on the definition of Fenchel conjugate for multivariables functions, we can obtain that

(11) f∗(λ(1),λ(2)) =max⁡(ω(1),ω(2))⁡(〈λ(1),ω(1)〉+〈λ(2),ω(2)〉−f(ω(1),ω(2))) =max⁡(ω(1),ω(2))⁡(〈λ(1),ω(1)〉+〈λ(2),ω(2)〉      −max⁡α∈[0,1]⁡α(b−(〈ω(1),x(1)〉−〈ω(2),x(2)〉))) =max⁡(ω(1),ω(2)) min⁡α∈[0,1]⁡(〈λ(1),ω(1)〉+〈λ(2),ω(2)〉        −α(b−(〈ω(1),x(1)〉−〈ω(2),x(2)〉))) =min⁡α∈[0,1]max⁡(ω(1),ω(2))⁡(−αb+〈λ(1)+αx(1),ω(1)〉        +〈λ(2)−αx(2),ω(2)〉) =min⁡α∈[0,1]⁡(−αb+max⁡ω(1)〈λ(1)+αx(1),ω(1)〉     +max⁡ω(2)〈λ(2)−αx(2),ω(2)〉).

Since the previous third equality follows from the strong max-min property, it can be transferred into a min-max problem. If ***λ*** ^(1)^ + *α* **x** ^(1)^ ≠ 0, max⁡_**ω**^(1)^_〈***λ*** ^(1)^ + *α* **x** ^(1)^, **ω** ^(1)^〉 is *∞*, and if ***λ*** ^(2)^ − *α* **x** ^(2)^ ≠ 0, max⁡_**ω**^(2)^_〈***λ*** ^(2)^ − *α* **x** ^(2)^, **ω** ^(2)^〉 is *∞*; otherwise, if ***λ*** ^(1)^ + *α* **x** ^(1)^ = 0 and ***λ*** ^(2)^ − *α* **x** ^(2)^ = 0, we have f*(***λ*** ^(1)^, ***λ*** ^(2)^) = −*αb*.

Back to the primal problem, we want to get a sequence of boundary **ω**_0_^(1,2)^, **ω**_1_^(1,2)^,…, **ω**_*T*_^(1,2)^  (**ω**_*t*_^(1,2)^ = (**ω**_*t*_^(1)^, **ω**_*t*_^(2)^), *t* ∈ {0,1,…, *T*}) which makes *J*(**ω**_0_^(1,2)^) ≥ *J*(**ω**_1_^(1,2)^) ≥ ⋯≥*J*(**ω**_*T*_^(1,2)^). Unfortunately, decreasing the objective function *J*(**ω** ^(1)^, **ω** ^(2)^) directly is impossible, while the training examples arrive in a steam. In order to avoid this contradiction, we propose a Fenchel conjugate transform for multi-view S^2^L problems based on coregularization.

An equivalent problem of (6) is

(12) min⁡ω0(1,2),ω1(1,2),…,ωT(1,2) ⁡12γ1||ω0(1)||2+12γ2||ω0(2)||2+∑t=1Tgt(ωt(1,2)),  s.t.     ∀i∈1,2,…,T,ωi(1)=ω0(1),ωi(2)=ω0(2).

Using the Lagrange dual function, we can rewrite (12) by introducing a vector group (***λ***_1_^(1,2)^, ***λ***_2_^(1,2)^,…, ***λ***_*T*_^(1,2)^):

(13) max⁡λ1(1,2),λ2(1,2),…,λT(1,2) ⁡min⁡ω0(1,2),ω1(1,2),…,ωT(1,2)⁡(12γ1||ω0(1)||2+12γ2||ω0(2)||2             +∑t=1Tgt(ωt(1,2))            +∑t=1T〈λ1(1),ω0(1)−ωt(1)〉            +∑t=1T〈λ1(2),ω0(2)−ωt(2)〉).

Consider the dual function

(14) D(λ1(1,2),λ2(1,2),…,λT(1,2))  =min⁡ω0(1,2),ω1(1,2),…,ωT(1,2)⁡(12γ1||ω0(1)||2+12γ2||ω0(2)||2          +∑t=1Tgt(ωt(1,2))          +∑t=1T〈λt(1),ω0(1)−ωt(1)〉          +∑t=1T〈λt(2),ω0(2)−ωt(2)〉)  =−max⁡ω0(1)⁡(∑t=1T〈−λt(1),ω0(1)〉−12γ1||ω0(1)||2)   −max⁡ω0(2)⁡(∑t=1T〈−λt(2),ω0(2)〉−12γ2||ω0(2)||2)   −∑t=1Tmax⁡λt(1,2)(〈λt(1),ωt(1)〉+〈λt(2),ωt(2)〉−gt(ωt(1,2)))  =−12γ1(−∑t=1Tλt(1))2   −12γ2(−∑t=1Tλt(2))2−∑t=1Tgt∗(λt(1,2)),

where *g*_t_*(***λ***_*t*_^(1,2)^) is the Fenchel conjugate of *g*_*t*_(**ω**_*t*_^(1,2)^). The primal problem can be described as maximizing the dual function as in the following

(15) min⁡ω(1,2) ⁡J(ω(1,2))=max⁡λ1(1,2),λ2(1,2),…,λT(1,2)D(λ1(1,2),λ2(1,2),…,λT(1,2)).

Based on our definition of Fenchel conjugate for multivariables functions, the Fenchel conjugate of *g*_*t*_(**ω**_*t*_^(1,2)^) can be rewritten as (based on Proposition 1 and Lemma A.2 in the appendix)

(16) gt∗(λt(1,2))  ={−σt(αt1+μαt2),if  λt(1)  =  −σtytαt1xt(1)−(1−σt)αt0γcxt(1),λt(2)  =  −σtμytαt2xt(2)+(1−σt)αt0γcxt(2),where  αt1,αt2∈[0,1],αt0∈[−1,1];∞,otherwise.  

Since our goal is to maximize the dual function, we can restrict to the first case in (16). *g*_t_*(***λ***_*t*_^(1,2)^) has 3 associated coefficient variables which are *α*_*t*0_, *α*_*t*1_, and *α*_*t*2_.

Based on the previous analysis, the dual function can be rewritten using a new coefficient vectors group **α**_1_, **α**_2_,…, **α**_*T*_, where **α**_*t*_ = [*α*_*t*0_, *α*_*t*1_, *α*_*t*2_]  (*t* ∈ {1,2,…, *T*}). Consider the following:

(17) D(α1,α2,…,αT) =−12γ1(∑t=1T(σtytαt1xt(1)+(1−σt)αt0γcxt(1)))2  −12γ2(∑t=1T(σtμytαt2xt(2)−(1−σt)αt0γcxt(2)))2  +∑t=1Tσt(αt1+μαt2).

As shown in (17), our task has been transferred to a constrained quadratic programming (QP) optimization problem. Every input training example brings a vector **α** into the dual function. **α**_1_, **α**_2_,…, **α**_*T*_ are independent, so we can update the vectors group (**α**_1_, **α**_2_,…, **α**_*T*_) on each learning round to ascend the dual problem incrementally. Obviously, unobserved examples would make no influence on the value of dual function in (17) by setting their associated coefficient variables to zero.

Denote (**α**_*i*_)_*t*_ the coefficient vector **α**_*i*_ on round *t* (*i* ∈ {1,2,…, *T*}). The update process of coefficient vectors group (**α**_1_, **α**_2_,…, **α**_*T*_) on round *t* should satisfy the following conditions:

- if *t* + 1 ≤ *i* ≤ *T*, (*α*_*i*0_)_*t*_, (*α*_*i*1_)_*t*_, (*α*_*i*2_)_*t*_ = 0;
- *D*((**α**_1_)_*t*_, (**α**_2_)_*t*_,…, (**α**_*T*_)_*t*_) ≥ *D*((**α**_1_)_*t*−1_, (**α**_2_)_*t*−1_,…, (**α**_*T*_)_*t*−1_).

The first one means that the unobserved examples do not make influence on the value of dual function, and the second means that the value of dual function never decreases during the online coregularization process. Therefore, the dual function on round *t* can also be written as

(18) D((α1)t,(α2)t,…,(αT)t) =−12γ1(∑i=1t(σiyi(αi1)txi(1)+(1−σi)(αi0)tγcxi(1)))2  −12γ2(∑i=1t(σiyiμ(αi2)txi(2)−(1−σi)(αi0)tγcxi(2)))2  +∑i=1tσi((αi1)t+μ(αi2)t).

Based on Lemmas A.1 and A.3 in the appendix, we can obtain that each coefficient vectors group (**α**_1_, **α**_2_,…, **α**_*T*_) has an associated boundary vector group (**ω** ^(1)^, **ω** ^(2)^). On round *t*, the associated boundaries of ((**α**_1_)_*t*_, (**α**_2_)_*t*_,…, (**α**_*T*_)_*t*_) are

(19) ωt(1)=1γ1∑i=1t(σiyi(αi1)txi(1)+(1−σi)(αi0)tγcxi(1)),ωt(2)=1γ2∑i=1t(σiμyi(αi2)txi(2)−(1−σi)(αi0)tγcxi(2)).

To make a summary, we propose a template online coregularization algorithm by dual ascending procedure in Algorithm 1.

Essentially, our online coregularization framework aims to break the large QP in the primal objective function into a series of dual ascending procedures on each learning round. Therefore, we can ascend the dual function in an online manner.

## 4. Analysis of Previous Work Based on Gradient Ascent in the Dual

In the previous section, a template algorithm framework for online coregularization is proposed based on the idea of ascending the dual function. In Algorithm 1, we can obtain that algorithms that derive from our framework may vary in one of two ways. First, different algorithms may update different dual variables on each learning round. The second way in which different algorithms may vary is how to update the chosen variables to ascend the dual function.

Some online coregularization algorithms [9, 10] have been suggested in recent years. These approaches have a similar idea of “*defining an instantaneous coregularized risk to avoid optimizing the primal coregularization problem directly.”* In these works, there are two popular instantaneous coregularized risk functions *J*_*t*_(**ω** ^(1)^, **ω** ^(2)^) which are defined as

(20) Jt(ω(1),ω(2)) =σt([1−yt〈ω(1),xt(1)〉]++μ[1−yt〈ω(2),xt(2)〉]+)  +(1−σt)γcd(〈ω(1),xt(1)〉,〈ω(2),xt(2)〉),Jt(ω(1),ω(2)) =12γ1||ω(1)||2+12γ2||ω(2)||2  +σt([1−yt〈ω(1),xt(1)〉]++μ[1−yt〈ω(2),xt(2)〉]+)  +(1−σt)γcd(〈ω(1),xt(1)〉,〈ω(2),xt(2)〉).

The online coregularization process in these works is based on convex programming with gradient descent on instantaneous coregularized risk function in kernel space. The step size *ρ*_*t*_ is often defined to decay at a certain rate [11], for example, $\rho_{t}=1/\sqrt{t}$. The update process in these approaches can be summarized as

(21) ωt(1,2)  =  ωt−1(1,2)  −  ρt∂Jt(ω(1,2))∂ω(1,2)|ωt−1(1,2).

In the following, we demonstrate that these algorithms can be derived from our online coregularization framework. Since the dual coefficient vectors **α**_1_, **α**_2_,…, **α**_*T*_ are independent, the dual function can be ascended by updating only the associated coefficient vector **α**_*t*_ of the new arrived training example (**x**_*t*_^(1,2)^, *y*_*t*_, *σ*_*t*_) on round *t* that means

(22) (αi)t  =  (αi)t−1, if  i≠t.

And the task on round *t* can be rewritten as ascending

(23) Dt(αt) =−12γ1(γ1ωt−1(1)  +  σtytαt1xt(1)  +  (1−σt)αt0γcxt(1))2  −12γ2(γ2ωt−1(2)  +  σtμytαt2xt(2)−(1−σt)αt0γcxt(2))2  +∑i=1tσi((αi1)t+μ(αi2)t).

Using a gradient ascent (GA) step on **α**_*t*_, the update process on round *t* can be written as

(24) (αt0)t=ρt∂Dt(αt)∂αt0|αt=0=−ρt(1−σt)γc(〈ωt−1(1),xt(1)〉−〈ωt−1(2),xt(2)〉),(αt1)t=ρt∂Dt(αt)∂αt1|αt=0=ρtσt[1−yt〈ωt−1(1),xt(1)〉]+,(αt2)t=ρt∂Dt(αt)∂αt2|αt=0=ρtσtμ[1−yt〈ωt−1(2),xt(2)〉]+,

where *ρ*_*t*_ ≥ 0 is a step size. 

In fact, the dual coefficient vectors in **ω**_*t*−1_^(1,2)^ can also be updated in (23). Since **ω**_*t*−1_^(1,2)^ has *t* − 1 dual coefficient vectors, it is impossible to update them, respectively. We introduce a new variable *η*_*t*_ into (23),

(25) Dt(αt,ηt) =−12γ1(γ1(1−ηt)ωt−1(1)  +  σtytαt1xt(1)     +(1−σt)αt0γcxt(1))2  −12γ2(γ2(1−ηt)ωt−1(2)  +  σtμytαt2xt(2)     −(1−σt)αt0γcxt(2))2  +∑i=1t−1σi((1−ηt)(αi1)t+μ(1−ηt)(αi2)t)  +σi((αt1)t+μ(αt2)t).

From (25), we can get that a gradient ascent update on *η*_*t*_ actually means to multiply all the dual coefficient vectors in **ω**_*t*−1_^(1,2)^ by 1 − *η*_*t*_. Since every dual coefficient variable in **ω**_*t*−1_^(1,2)^ is constrained, we also constrain *η*_*t*_ ∈ [0,1]. The initial value of *η*_*t*_ is zero. While using a gradient ascent on *η*_*t*_, we obtain that

(26) ηt=ρt[γ1〈ωt−1(1),ωt−1(1)〉+γ2〈ωt−1(2),ωt−1(2)〉   −∑i=1t−1σi(αi1)t−∑i=1t−1σiμ(αi2)t]+.

Based on the previous analysis, the gradient ascent process of boundary vector group (**ω** ^(1)^, **ω** ^(2)^) can be written as

(27) ωt(1)=(1−ηt)ωt−1(1)+1γ1(σtyt(αt1)txt(1)+(1−σt)(αt0)tγcxt(1)),ωt(2)=(1−ηt)ωt−1(2)+1γ2(σtμyt(αt2)txt(2)−(1−σt)(αt0)tγcxt(2)).

As far as we know, all the existing online coregularization algorithms in previous work can be viewed as an approximation of our dual ascending process using gradient ascent in (27). 

## 5. Deriving New Algorithms Based on Aggressive Dual Ascending (ADA) Procedures

In the previous section, we show that the online coregularization algorithms in previous work can be derived from our framework. These algorithms lead to a conservative increase of the value of the dual function since they only modify a single dual vector using gradient ascent on each learning round. In fact, more aggressive online coregularization algorithms can also be derived from our framework. 

In this section we describe broader and, in practice, more powerful online coregularization algorithms which increase the dual function more aggressively on each learning round. The motivation for the new algorithms is as follows. Intuitively, update schemes that yield larger increases of the dual function are likely to reach the minimal value of primal objective function faster. Thus, they are in practice likely to suffer a smaller number of mistakes. 

### 5.1. Updating Single Dual Coefficient Vector

The update scheme we described in Section 4 for increasing the dual function modifies the associated coefficient vector of the new arrived training example which is based on gradient ascent, and all the variables in the vector share a same step size. This simple algorithm can be enhanced by solving the following optimization problem on each learning round

(28) max⁡αt1,αt2∈[0,1],αt0∈[−1,1] ⁡D(α1,α2,…,αT)   s.t.     ∀i≠t,αi  =  (αi)t−1.

According to the type of the new arrived example, (28) can be solved in different ways. If the new arrived example is labeled, we have *σ*_*t*_ = 1, and the task on round *t* can be rewritten as

(29) max⁡αt1,αt2∈[0,1]⁡−12γ1(γ1ωt−1(1)  +  ytαt1xt(1))2  −12γ2(γ2ωt−1(2)  +  μytαt2xt(2))2+(αt1+μαt2) =max⁡αt1∈[0,1]⁡(−12γ1(γ1ωt−1(1)  +  ytαt1xt(1))2+αt1)  +max⁡αt2∈[0,1]⁡(−12γ2(γ2ωt−1(2)  +  μytαt2xt(2))2+μαt2).

Since *α*_*t*1_, *α*_*t*2_ ∈ [0,1], we can obtain that

(30) (αt1)t=min⁡⁡{γ1[1−yt〈ωt−1(1),xt(1)〉]+〈xt(1),xt(1)〉,1},(αt2)t=min⁡⁡{γ2[1−yt〈ωt−1(2),xt(2)〉]+μ〈xt(2),xt(2)〉,1}.

Otherwise, if the new arrived example is unlabeled, we have *σ*_*t*_ = 0, and (28) can be rewritten as

(31) max⁡αt0∈[−1,1]⁡−12γ1(γ1ωt−1(1)  +  αt0γcxt(1))2   −12γ2(γ2ωt−1(2)  −  αt0γcxt(2))2.

Since *α*_*t*0_ ∈ [−1,1], we can obtain that

(32) (αt0)t ={min⁡⁡{−(1/γc)(〈ωt−1(1),xt(1)〉−〈ωt−1(2),xt(2)〉)(1/γ2)〈xt(2),xt(2)〉+(1/γ1)〈xt(1),xt(1)〉,1},if  (αt0)t≥0max⁡⁡{−(1/γc)(〈ωt−1(1),xt(1)〉−〈ωt−1(2),xt(2)〉)(1/γ2)〈xt(2),xt(2)〉+(1/γ1)〈xt(1),xt(1)〉,−1}if  (αt0)t<0.

Based on the previous analysis, the update process of boundary vector group (**ω** ^(1)^, **ω** ^(2)^) can be written as

(33) ωt(1)=ωt−1(1)  +  1γ1 ×(σtyt(αt1)txt(1)+(1−σt)(αt0)tγcxt(1)),ωt(2)=ωt−1(2)  +  1γ2 ×(σtμyt(αt2)txt(2)−(1−σt)(αt0)tγcxt(2)).

In contrast to the gradient approaches in Section 4, this approach ascends the dual function more aggressively. So far, our focus was on an update which modifies a single dual coefficient vector. In fact, all the associated coefficient vectors of the arrived examples can be updated during the online regularization process. We now examine another update scheme based on our online coregularization framework that suggests the modification of multiple dual coefficient vectors on each learning round.

### 5.2. Updating Multiple Dual Coefficient Vectors

We now describe a new update scheme which modifies multiple dual coefficient vectors on each learning round. In practice, we can get the example set {(**x**_1_^(1,2)^, *y*_1_, *σ*_1_), (**x**_2_^(1,2)^, *y*_2_, *σ*_2_),…, (**x**_*t*_^(1,2)^, *y*_*t*_, *σ*_*t*_)} on round *t*, and all the associated coefficient vectors of these examples can be updated to ascend the dual function. 

Denote *S*_*t*_⊆[*t*] the buffer on round *t*, where *t* ∈ *S*_*t*_ and [*t*] = {1,2,…, *t*}. If *i* ∈ *S*_*t*_, the associated coefficient vector of the *i*th example can be updated on round *t*. We optimize the dual function over the dual coefficient vectors whose indices belong to *S*_*t*_. Consider the following:

(34) max⁡αi(i∈St)⁡ D(α1,α2,…,αT) s.t.  ∀i∉St,αi=(αi)t−1.

Obviously, this more general update scheme could make a larger increase on each learning round. If setting *S*_*t*_ = [*t*], (34) makes use of all the examples that have been observed and thus is likely to make the largest increase of the dual function. And if setting *S*_*t*_ = {*t*}, (34) degenerates in the update scheme we have described in Section 5.1. The time complexity for solving (34) is *O*((2×size  of  (*S*_*t*_))^3^) (worst case).

Similar to the idea in Section 4, we can also introduce a new variable *η*_*t*_ into (34),

(35) max⁡αi(i∈St),ηt∈[0,1] ⁡D(α1,α2,…,αT)  s.t.   ∀i∉St,αi=(1−ηt)×(αi)t−1.

*η*_*t*_ is more like a *forgetting factor* [21] to downweight the contribution of observations whose indices do not belong to *S*_*t*_. When tracking the changes in the data stream, it is likely that recent observations will be more indicative of its appearance than more distant ones. Incorporating a *forgetting factor *in the online learning algorithms is a good way to moderate the balance between old and new observations. We can also obtain that *η*_*t*_ = 0 (*t* ∈ {1,2,…, *T*}) indicates that no forgetting is to occur.

For practical purpose, we test two choices of *S*_*t*_ to update multiple dual coefficient vectors in this paper.

- Buffer-*N*. Let the buffer size be *τ*. Buffer-*N* replaces the oldest example (**x**_*t*−*τ*_^(1,2)^, *y*_*t*−*τ*_, *σ*_*t*−*τ*_) in the buffer with the new arrived example (**x**_*t*_^(1,2)^, *y*_*t*_, *σ*_*t*_) on each learning round, which means *S*_*t*_ = {*t* − *τ* + 1, *t* − *τ* + 2,…, *t*}.
- Buffer-*L*. This buffering strategy replaces the oldest unlabeled point in the buffer with the incoming point while keeping labeled points. The oldest labeled point is evicted from the buffer only when it is filled with labeled points.

**Figure 2:**
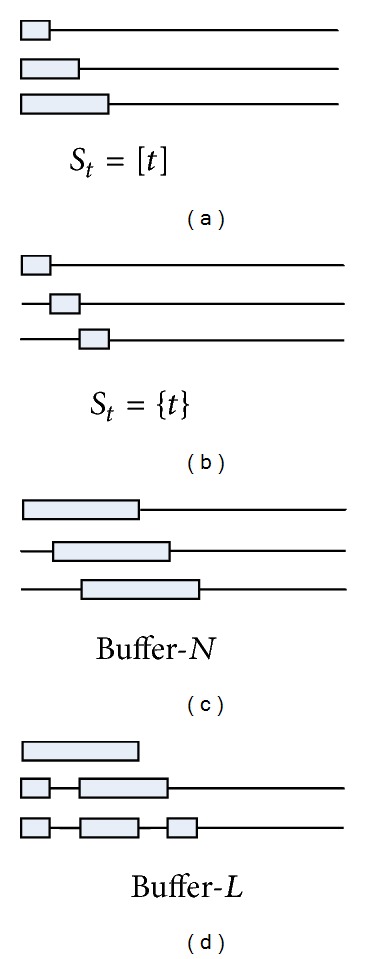
Four choices of *S*_*t*_ to update multiple dual coefficient vectors. The horizontal thin line on each learning round represents the whole training data sequence, while the thick boxes represent the set of examples whose dual coefficient vectors are used to ascend the dual function on that round. Essentially, different choices of *S*_*t*_ construct different QP problems on each learning round. Obviously, if *S*_*t*_ = [*t*], the update scheme chooses to solve the largest possible QP on each learning round; and if *S*_*t*_ = {*t*}, the update scheme chooses to solve the smallest possible QP on each learning round.

Figure 2 shows the set of the associated coefficient vectors which are used to ascend the dual function on each learning round for different choices of *S*_*t*_. Essentially, different choices of *S*_*t*_ construct different QP problems on each learning round.

### 5.3. Sparse Approximations for Kernel Representation

In practice, kernel functions are always used to find a linear classifier, like SVM. Our online coregularization framework only contains the product of two points, so we can easily introduce the kernel function into our framework. If we note *K* the kernel matrix such that

(36) Kij=Φ(xi)·Φ(xj),

**x**_*i*_ can be replaced by Φ(**x**_*i*_) in our framework. Therefore, we can rewrite (19) as

(37) ωt(1)=1γ1∑i=1t(σiyi(αi1)t+(1−σi)(αi0)tγc)Φ(1)(xi(1))=∑i=1t(βi(1))tΦ(1)(xi(1)),ωt(2)=1γ2∑i=1t(σiμyi(αi2)t−(1−σi)(αi0)tγc)Φ(2)(xi(2))=∑i=1t(βi(2))tΦ(2)(xi(2)).

Unfortunately, our previous derived online coregularization algorithms with kernel functions have to store the example sequence up to the current round, and the stored matrix size is *t* × *t* (worst case). For practical purpose, we present two approaches to sparsify the kernel representation of boundaries on each learning round. 

*Absolute Threshold.* To construct a sparse representation for the boundaries, absolute threshold discards the examples whose associated coefficients *β* ^(1,2)^ are close to zero. Let *ε* > 0 denote the absolute threshold. When an arrived example **x**_*i*_^(1,2)^ would not be used to update the boundaries in further learning process, **x**_*i*_^(1,2)^ is discarded if |(*β*_*i*_^(1,2)^)_*t*_| < *ε*. The examples whose indices are in *S*_*t*_ cannot be discarded on round *t* since they would be used to ascend the dual function.

*k Maximal Coefficients *(*k-MC*). Another way to sparsify the kernel representation is to keep the examples of which the absolute value of *β* ^(1,2)^ is the first *k* maximum. Similar as the absolute threshold, *k*-MC does not discard the examples in *S*_*t*_ which would be used to ascend the dual function on round *t*. Based on this sparse approximation, the stored matrix size on round *t* reduces to (*k* + size  of  (*S*_*t*_)) × (*k* + size  of  (*S*_*t*_)).

The previous two sparse approximations are both motivated by the fact that the examples which have larger coefficients tend to exert more influence on our learned boundaries.

## 6. Experiments

This section presents a series of experimental results to report the effectiveness of our derived online coregularization algorithms. It is known that the performance of semi-supervised learning depends on the correctness of model assumptions. Thus, our focus is on comparing different online coregularization algorithms with multiple views, rather than different semi-supervised regularization methods.

We report experimental results on two synthetic and a real word binary classification problems. The prediction function in online coregularization algorithms are adopted as the average of the prediction functions from two views

(38) ft(x(1,2))=sign⁡(12(〈ωt(1),x(1)〉+〈ωt(2),x(2)〉)).

Based on the idea of “*interested in the best performance and simply select the parameter values minimizing the error*” [3], we select combinations of the parameter values on a finite grid in Table 1, and it is sufficient to perform algorithm comparisons. 

The training sequences are generated randomly from each data sets. To avoid the influence of different training sequences, all results on each dataset are the average of five such trials (this idea is inspired by [11]). The error rates have ±1 standard deviation. While using buffering strategies to update multiple dual coefficient vectors, the buffer size is fixed at 100 to avoid high computational complexity on each learning round. We implemented all the experiments using MATLAB. 

### 6.1. Two-Moons-Two-Lines Synthetic Data Set

**Figure 3:**
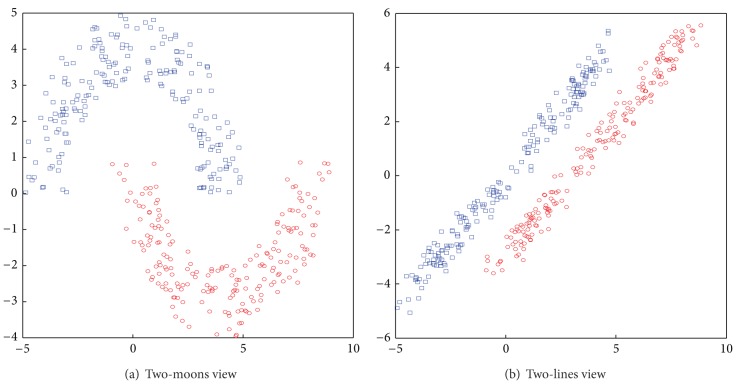
Distribution of two-moons-two-lines data set.

This synthetic data set is generated similarly to the toy example used in [16, 19] in which examples in two classes appear as two moons in one view and two oriented lines in another (see Figure 3 for an illustration). This data set contains 2000 examples, and only 5 examples for each class are labeled. A Gaussian and linear kernel are chosen for the two-moons and two-lines views respectively. In this data set, the offline coregularization algorithms (CoLapSVM) [16] achieve an error rate of 0.

The best performance of all the online coregularization algorithms in Section 5 is presented in Table 2. We also provide some additional details during the online coregularization process. 

**Figure 4:**
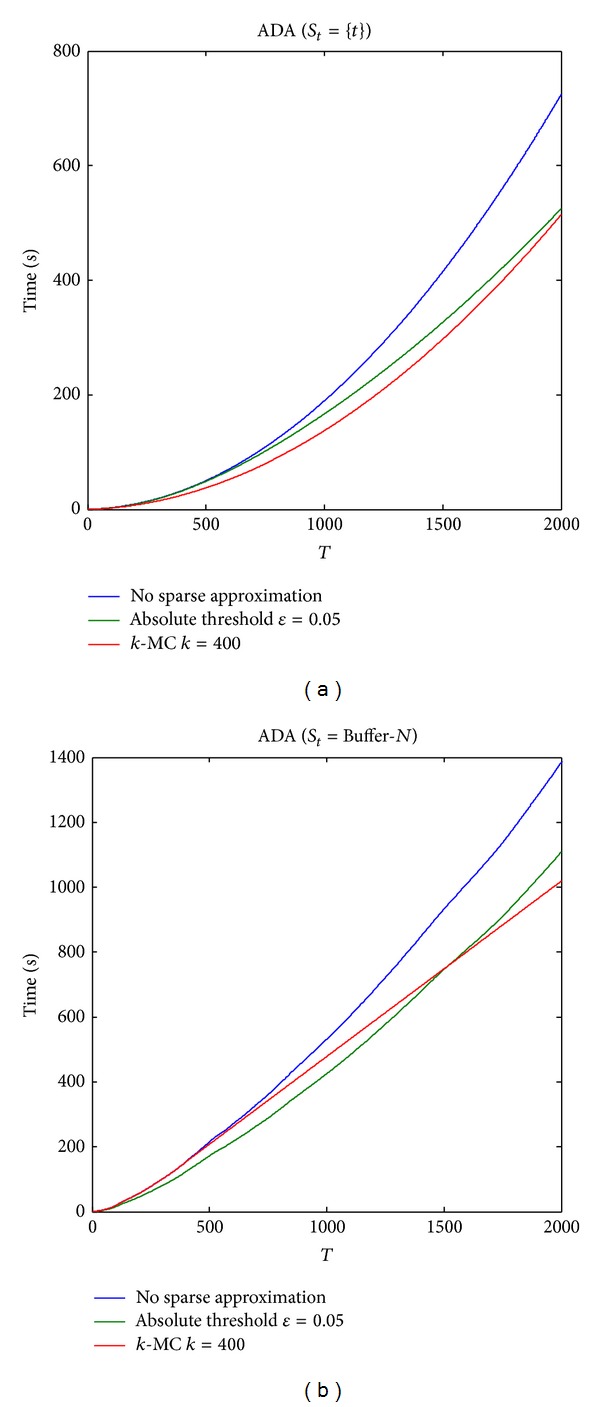
Cumulative runtime growth curves. From left to right (a) ADA (*S*_*t*_ = {*t*}); (b) ADA (Buffer-*N*). The curves have the similar trends for different online coregularization algorithms. Online coregularization algorithms with sparse representation perform better than the basic online coregularization algorithms on the growth rate.

We compare cumulative runtime curves of online coregularization algorithms with different sparse approximation approaches in Figure 4. Online coregularization algorithms with sparse representation perform better than the basic online coregularization algorithms on the growth rate. The cumulative runtime growth curves of online coregularization algorithms with sparse approximation approaches scale only linearly, while the others scale quadratically.

**Figure 5:**
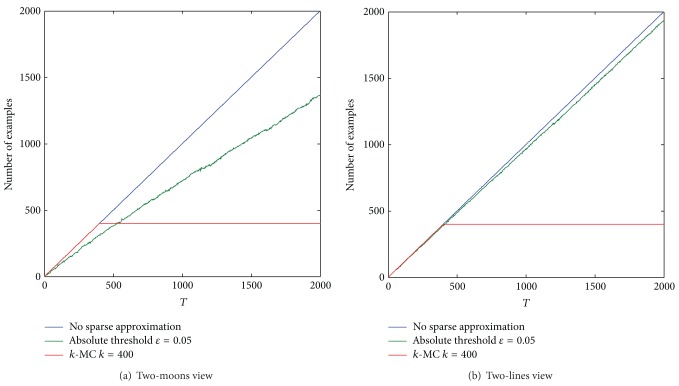
The number of examples in the kernel representation of boundary vectors in two views for different sparse approximation approaches. The online coregularization algorithm used in this experiment is ADA (Buffer-*N*). If no sparse representation approaches are used, the kernel representation contains all the arrived examples on each learning round. The number of examples in the kernel representation increases slowly while using an absolute threshold, and the number is at most *k* while using *k*-MC for online coregularization algorithms.

We also compare the number of examples in the kernel representation of boundary vectors in two views on each learning round for different sparse approximation approaches.  Figure 5 shows that only part of examples have to be stored (and computed) while using sparse approximation approaches. Online coregularization algorithms without sparse approximation approaches are time consuming and memory-consuming, and it is intractable to apply them to real-world long time tasks.

**Figure 6:**
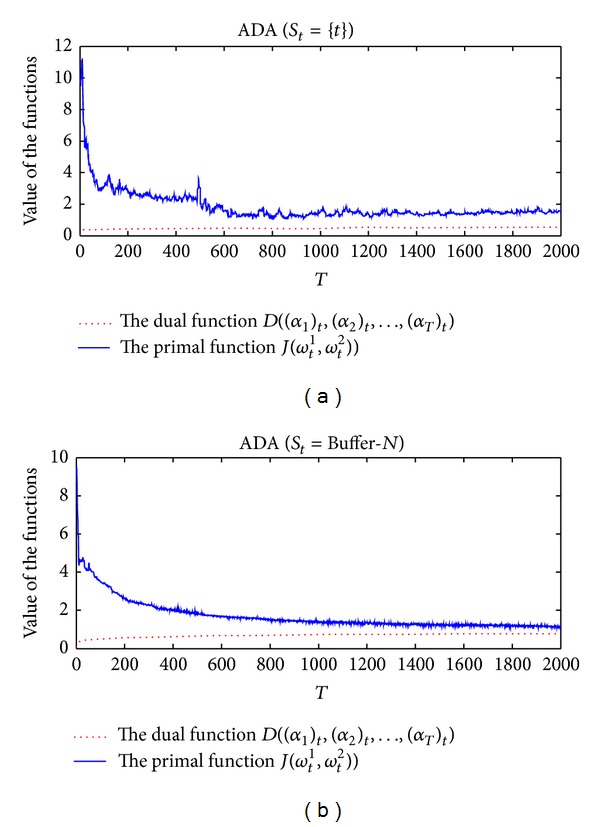
The value curves of the primal objective function and the dual function during the incremental learning process. From left to right (a) ADA (*S*_*t*_ = {*t*}) and (b) ADA (Buffer-*N*). The two curves approach each other as *t* increases.

In Section 3, we have demonstrated the relationship between the primal objective function *J*(**ω**_*t*_^(1,2)^) and the dual function *D*((**α**_1_)_*t*_, (**α**_2_)_*t*_,…, (**α**_*T*_)_*t*_). We compare the primal objective function versus the dual function on the training sequence of two-moons-two-lines data set as *t* increases in Figure 6. The result shows that the two curves approach each other along the online coregularization algorithms run. The value of dual function never decreases as *t* increases; correspondingly, the curve of primal function has a downward trend and some little fluctuations. We also observe that the curve of the primal objective function which updates multiple dual coefficient vectors on each learning round shows a more smooth downward trend and has less rapid fluctuations. This experiment supports the theory that increasing the dual problem can achieve comparable risks of the primal objective function. 

**Figure 7:**
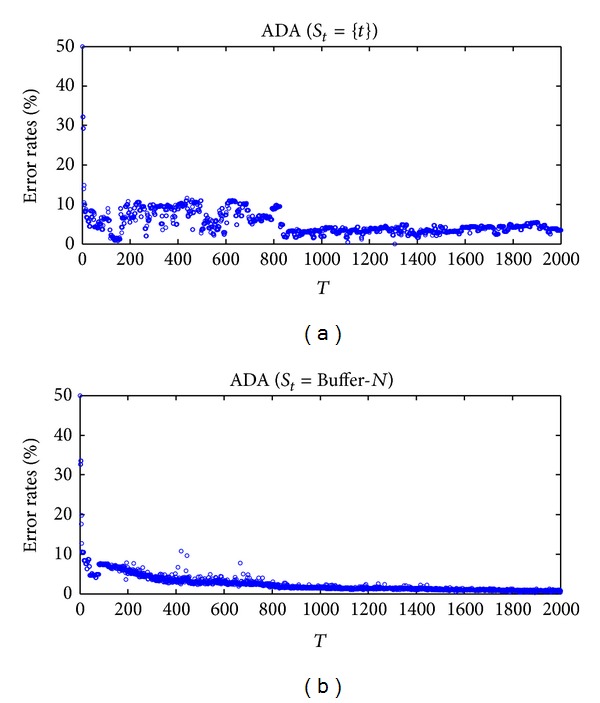
Error rates of **ω**_*t*_^(1,2)^ on the whole two-moons-two-lines data set. From left to right (a) ADA (*S*_*t*_ = {*t*}) and (b) ADA (Buffer-*N*). The error rate of **ω**_*t*_^(1,2)^ has a downward trend, but it is not always decreasing during the online coregularization process.

We report the performance of **ω**_*t*_^(1,2)^ on the whole two-moons-two-lines data set in Figure 7. This result shows that the boundary vector is adjusted to be a better one along the online coregularization algorithms run. Since our algorithms adjust the decision boundary vector according to the local agreement of *S*_*t*_ in the two views on each learning round, the curve of the error rate is not always decreasing along the online coregularization process. It is also the reason why our online coregularization algorithms can track the changes in the data sequence (more detail in Section 6.3). Similar as the experiments in Figure 6, we can observe that the error rate of **ω**_*t*_^(1,2)^ which updates multiple dual coefficient vectors on each learning round shows a more smooth downward trend and has less rapid fluctuations.

### 6.2. Web Page Categorization

We applied our derived online coregularization algorithms to the WebKB text classification task studied in Blum and Mitchell [2]; Sindhwani et al. [16]; Sun and Shawe-Taylor [19]. The task is to predict whether a web page is a course home page or not. The data set consists of 1051 web pages in two views (page and link) collected from the computer science department web sites at four U.S. universities: Cornell, University of Washington, University of Wisconsin, and University of Texas. The first view of the data is the textual content of a webpage itself, and the second view is all links pointing to the web page from other web pages. We preprocessed each view by removing stop words, punctuation, and numbers and then applied Porter's stemming to the text [22]. This problem has an unbalanced class distribution since there are a total of 230 course home pages and 821 noncourse. In addition, words that occur in five or fewer documents were ignored. This resulted in 2332 and 87 dimensional vectors for two views, respectively. Finally, document vectors were normalized to TDIDF features (the product of term frequency and inverse document frequency) [23]. As in [19], we randomly label 3 course and 9 noncourse examples for each class. In this experiment, the linear kernel is used for both views.

In this data set, the offline coregularization algorithms (CoLapSVM) [16] achieve an error rate of 6.32%. In Table 3, we report the best performance of all the online coregularization algorithms on the web page data set.

### 6.3. Rotating Two-Moons-Two-Lines Synthetic Data Set

**Figure 8:**
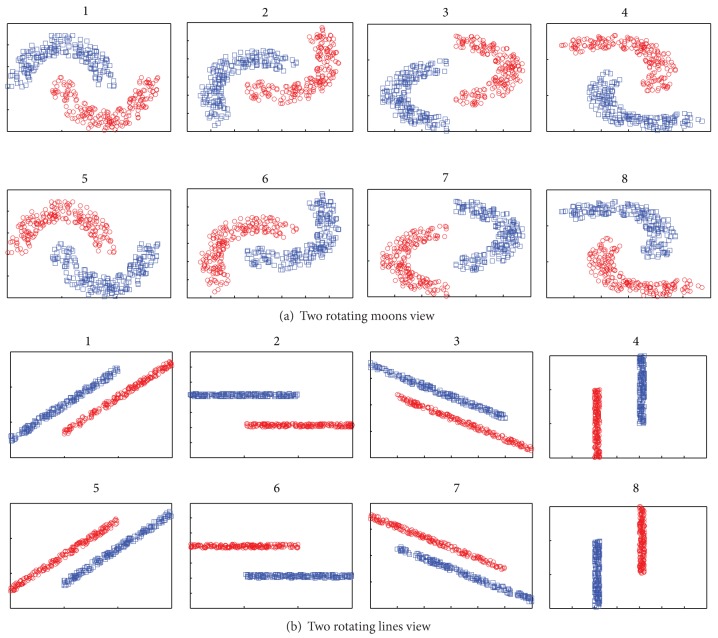
Rotating two-moons-two-lines data sequence. We spin the two-moons-two-lines data set in top left of the two views during the sequence so that two moons and two lines smoothly rotate 360° in every 8000 examples.

When the underlying distributions, both *P*(**x** ^(1,2)^) and *P*(*y* | **x** ^(1,2)^), change during the course of learning, the algorithms are expected to track the changes in the data sequence. In this subsection, we test the applicability of our framework to settings where the target hypotheses in different views are not fixed but rather drift with the sequence of examples. To demonstrate that our online coregularization algorithms can handle concept drift, we perform our experiments on rotating two-moons-two-lines data sequence. This data set contains 8000 examples, and only 1% examples for each class are labeled.  Figure 8 shows that the two-moons-two-lines data set smoothly rotate 360° during the sequence, and the target boundaries in two views drift with the sequence of examples. In the rotating two-moons-two-lines data set, the points will change their true labels during the sequence, and every stationary decision boundary will have an error rate of 50% approximately. A Gaussian and linear kernel are chosen for the two rotating moons and two rotating lines view, respectively.

In Table 4, we report the best performance of all the online coregularization algorithms on the rotating two-moons-two-lines data sequence. In this experiment, we also discuss the effect of the buffer size to track the changes in the data sequence.

**Figure 9:**
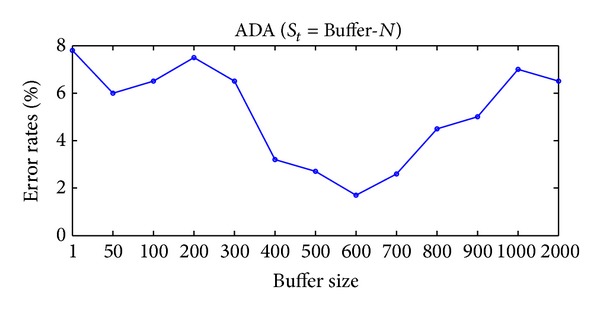
Error rates of ADA (Buffer-*N*) with different buffer sizes on the rotating two-moons-two-lines synthetic data sequence.

Obviously, when tracking the changes in the rotating two-moons-two-lines data sequence, it is likely that recent examples will be more indicative of the boundaries than more distant ones. Buffer-*N* updates the boundaries using the recent examples, it is also the reason why ADA (Buffer-*N*) performs better than the other online coregularization algorithms. We report the error rates of ADA (Buffer-*N*) with different buffer sizes on rotating two-moons-two-lines synthetic data sequence in Figure 9. This experiment illustrates that a suitable size of Buffer-*N* is able to adapt to the changing sequence and maintain a small error rate.

## 7. Conclusion and Further Discussion

In this paper we presented an online coregularization framework based on the notion of ascending the dual function. We demonstrated that the existing online coregularization algorithms in previous work can be viewed as an approximation of our dual ascending process using gradient ascent. New online coregularization algorithms are derived based on aggressive dual ascending procedures. For practical purpose, we proposed two sparse approximation approaches for kernel representation to reduce the computational complexity. Experiments showed that our online coregularization algorithms can adjust the boundary vector with the input sequence and have risk and error rates comparable to offline algorithms. Specially, our online coregularization algorithms can handle the settings where the target boundaries are not fixed but rather drift with the sequence of examples in different views.

There are many interesting questions remaining in the online semi-supervised learning setting. For instance, we plan to study new online learning algorithms for other semi-supervised learning models. Another direction is how to choose effective combination of the parameter values more intelligently during the online coregularization process.

## Figures and Tables

**Algorithm 1:**
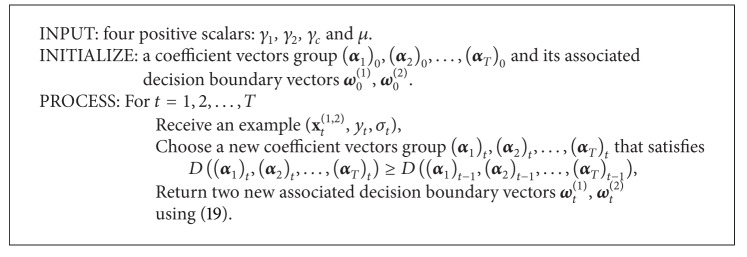
A template online co-regularization algorithm for multiview semi-supervised binary classification problems. This template algorithm aims for increasing the dual function on each learning round.

**Table 1:** A finite grid of parameter values. We find the best performance of each online co-regularization algorithm on this finite grid.

Parameter	Values
*γ*_1_	10^−3^, 10^−2^, 10^−1^, 1, 10^1^, 10^2^, 10^3^
*γ*_2_	10^−3^, 10^−2^, 10^−1^, 1, 10^1^, 10^2^, 10^3^
*γ*_*c*_	10^−3^, 10^−2^, 10^−1^, 1, 10^1^, 10^2^, 10^3^
*μ*	10^−3^, 10^−2^, 10^−1^, 1, 10^1^, 10^2^, 10^3^

**Table 2:** Mean test error rates on the two-moons-two-lines synthetic data set. The error rates are reported for three different sparse approximations. For gradient ascent, we choose a decaying step size $\rho_{t}=0.1/\sqrt{t}$. The result shows that our derived online co-regularization algorithms achieve test accuracy comparable to offline co-regularization (CoLapSVM). The online co-regularization algorithms based on aggressive dual ascending procedures perform better than those based on gradient ascent.

Online co-regularization algorithms	Gradient ascent	ADA *S*_*t*_ = {*t*}	ADA *S*_*t*_ = [*t*]	ADA Buffer-*N*	ADA Buffer-*L*
Error rates (%)					
No sparse approximation	12.15	8.95	3.95	4.25	4.05
Absolute threshold (*ε* = 0.05)	10.80	9.65	3.95	4.05	4.05
*k*-MC (*k* = 400)	13.30	9.85	3.95	7.00	5.35

**Table 3:** Mean test error rates on the web page data set. The error rates are reported for three different sparse approximations. For gradient ascent, we choose a decaying step size $\rho_{t}=0.1/\sqrt{t}$. The result shows that our derived online co-regularization algorithms achieve test accuracy comparable to offline co-regularization (CoLapSVM).

Online co-regularization algorithms	Gradient ascent	ADA *S*_*t*_ = {*t*}	ADA *S*_*t*_ = [*t*]	ADA Buffer-*N*	ADA Buffer-*L*
Error rates (%)					
No sparse approximation	10.94	11.89	7.52	8.47	7.80
Absolute threshold (*ε* = 0.05)	11.23	11.70	7.52	8.66	7.80
*k*-MC (*k* = 400)	11.32	11.41	7.80	8.75	7.99

**Table 4:** Mean test error rates on the rotating two-moons-two-lines synthetic data sequence. The error rates are reported for three different sparse approximations. For gradient ascent, we choose a stationary step size *ρ*_*t*_ = 0.1. The result shows that our derived online co-regularization algorithms are able to track the changes in the sequence and maintain a smaller error rate compared with batch learning algorithms. Specially, ADA (Buffer-*N*) performs better than the other online co-regularization algorithms.

Online co-regularization algorithms	Gradient ascent	ADA *S*_*t*_ = {*t*}	ADA Buffer-*N*	ADA Buffer-*L*
Error rates (%)				
No sparse approximation	12.75	7.75	6.43	20.35
Absolute threshold (*ε* = 0.05)	11.55	10.75	9.27	18.75
*k*-MC (*k* = 400)	11.55	10.35	9.45	18.35

## Appendix

### Fenchel Conjugate

The Fenchel conjugate of a function *f* : *S* → ℝ is defined as

(A.1) f∗(λ)=sup⁡{〈λ,ω〉−f(ω):ω∈S}.

Since *f** is defined as a supremum of linear functions, it is convex. Here, we describe a few lemmas of Fenchel conjugate which we use as theoretical tools in this paper. More details are in [24].

Lemma A.1 Let *f* be a closed and convex function and let ∂*f*(**ω**) be its differential set at **ω**, Then, for all ***λ*** ∈ ∂*f*(**ω**), we have *f*(**ω**) + *f**(***λ***) = 〈***λ***, **ω**〉.

Proof Since ***λ*** ∈ ∂*f*(**ω**) and *f* is closed, and convex, we know that *f*(**ω**′) − *f*(**ω**) ≥ 〈***λ***, **ω**′ − **ω**〉 for all **ω**′ ∈ *S*. Equivalently

(A.2) 〈λ,ω〉−f(ω)≥sup⁡{〈λ,ω′〉−f(ω′):ω∈S}.

The right-hand side of the previous equation equals to *f**(***λ***), and thus

(A.3) 〈λ,ω〉−f(ω)≥f∗(λ)→〈λ,ω〉−f∗(λ)≥f(ω).

The assumption that *f* is closed and convex implies that *f* is the Fenchel conjugate of *f**. Thus,

(A.4) f(ω)=sup⁡{〈λ′,ω〉−f(λ′):λ′∈S}≥〈λ,ω〉−f(ω).

Combining the two inequalities, we have

(A.5) f(ω)+f∗(λ)=〈λ,ω〉.

Lemma A.2 Let *f*(**ω**) = ∑_*i*=1_^*k*^[*b*_*i*_−〈**ω**,**x**_*i*_〉]_+_, where for all *i* ∈ {1,2,…, *k*}, *b*_*i*_ ∈ ℝ and **x**_*i*_ ∈ ℝ^*n*^. The Fenchel conjugate of *f*(**ω**) is

(A.6) f∗(λ) ={−∑i=1kαibi
if  λ∈{−∑i=1kαixi:∀i∈{1,2,…,k},αi∈[0,1]}∞otherwise.

Proof We first rewrite the *f*(**ω**) as follows:

(A.7) f(ω)=∑i=1k[bi−〈ω,xi〉]+=max⁡α1,α2,…,αk∈[0,1]⁡∑i=1kαi(bi−〈ω,xi〉),

where *α*_*i*_ ∈ [0,1] for all *i* ∈ {1,2,…, *k*}. Based on the definition of Fenchel conjugate, we can obtain that

(A.8) f∗(λ)=max⁡ω⁡(〈λ,ω〉−f(ω))=max⁡ω⁡(〈λ,ω〉−max⁡α1,α2,…,αk∈[0,1]⁡∑i=1kαi(bi−〈ω,xi〉))=max⁡ω min⁡α1,α2,…,αk∈[0,1]⁡(〈λ,ω〉−∑i=1kαi(bi−〈ω,xi〉))=min⁡α1,α2,…,αk∈[0,1] max⁡ω⁡(−∑i=1kαibi+〈λ+∑i=1kαixi,ω〉)=min⁡α1,α2,…,αk∈[0,1]⁡(−∑i=1kαibi+max⁡ω〈λ+∑i=1kαixi,ω〉).

Since the aforementioned third equality follows from the strong max-min property, it can be transferred into a min-max problem. If ***λ*** + ∑_*i*=1_^*k*^ *α*_*i*_ **x**_*i*_ ≠ 0, $\underset{\omega }{\max}\langle \lambda +\sum_{i=1}^{k}\alpha_{i}x_{i},\omega \rangle$ is *∞*; otherwise, if ***λ*** + ∑_*i*=1_^*k*^ *α*_*i*_ **x**_*i*_ = 0, we have *f**(***λ***) = −∑_*i*=1_^*k*^ *α*_*i*_ *b*_*i*_.

Lemma A.3 Let ||·|| be any norm on ℝ^**n**^, and let *f*(**ω**) = (1/2)||**ω**||^2^ with *S* = ℝ^**n**^. Then *f**(***λ***) = (1/2)||***λ***||_∗_^2^ where ||·||_∗_ is the dual norm of ||·||. The domain of *f** is also ℝ^**n**^. For example, if *f*(**ω**) = (1/2)||**ω**||_2_^2^ then *f**(***λ***) = (1/2)||***λ***||_2_^2^ since *ℓ*_2_ norm is dual to itself.

Lemma A.4 Let *f* be a function, and let *f** be its Fenchel conjugate. For *a* > 0 and *b* ∈ ℝ, the Fenchel conjugate of *g*(**ω**) = *af*(**ω**) + *b* is *g**(***λ***) = *af**(***λ***/*a*) − *b*.
